# Approach to the Pretransplant Evaluation of the Living Kidney Donor

**DOI:** 10.1155/2011/245738

**Published:** 2011-12-29

**Authors:** Mala Sachdeva, Madhu Bhaskaran, Ernesto P. Molmenti, Donna Dalton, Joseph Mattana

**Affiliations:** ^1^Division of Kidney Diseases and Hypertension, Department of Medicine, Hofstra North Shore-LIJ School of Medicine, Great Neck, NY 11021, USA; ^2^Department of Surgery, Hofstra North Shore-LIJ School of Medicine, Manhasset, NY 11030, USA

## Abstract

Evaluation of the potential kidney donor is a complex activity that differs substantially from other types of preoperative assessments. The well being of the donor, who derives no medical benefit from this surgery, must be assured in both the short term and long term, and the potential adverse consequences to the recipient must be determined as well. The criteria that must be met for a person to donate a kidney are rigorous and include medical, social, psychosocial, ethical, and legal issues. Donor evaluation can be divided into assessments to protect the health and safety of the donor and assessments to protect the health and safety of the recipient. This article provides an approach to evaluating a donor, focusing on the complex issues that an evaluator is faced with. A careful assessment of risks and benefits to both the donor and recipient can lead to favorable outcomes.

## 1. Introduction

A living kidney donor can improve the quality of life and offer a survival advantage to the recipient. Since 1998, as per Organ Procurement and Transplantation Network data, there have been approximately 309,319 kidney transplantations and of these approximately 108,150 are from living kidney donors [1]. The shortage of living kidney donors is one of the central issues that prolongs transplantation. This shortage could be due to the stringent criteria that must be applied to protect the health of the donor not only in the perioperative period but long term as well.

 The evaluation of a donor presents unique issues that are addressed in a far different manner compared to patients undergoing other types of surgeries where the risk/benefit assessment is of a completely different nature. A balance between doing no harm to the donor while doing good for the recipient must be achieved. This risk/benefit analysis is not always straightforward and may not be readily apparent to the nontransplant evaluator.

The standard preoperative clearance does not apply entirely to the kidney donor. The issues created by many donors can be complex and thought provoking and require thorough and detailed evaluations. Not only is a comprehensive review of their medical health necessary, but also a complete assessment of their social and psychosocial well being must be performed as well. In addition, ethical and legal issues must be taken into consideration. Aside from the general cardiovascular risk assessment which is needed for all donors as well as addressing immunological issues including blood typing and crossmatching, this article will focus on the multifaceted issues that arise as a donor is being evaluated, paying particular attention to the most common dilemmas and challenges that we face when evaluating them. These issues can be divided into two general categories: assessments to protect the health and safety of the donor and donor assessments to protect the health and safety of the recipient. Both can be further subdivided into medical, renal, lifestyle, and psychosocial issues. There is allowance of overlap amongst the subcategorized issues [Table 1].

## 2. Assessments to Protect the Health and Safety of the Donor

### 2.1. General Medical Issues

#### 2.1.1. Diabetes Mellitus

While diabetic patients and prediabetic patients regularly undergo surgery, the approach in the potential kidney donor is entirely different. One concern is that the presence of a single kidney in a diabetic patient may result in an accelerated decline in kidney function if diabetic nephropathy develops.

Most centers initiate screening with Hemoglobin A1C (A1C), and/or fasting blood glucose (FBG). If a person has any risk factors for developing diabetes mellitus or any abnormalities in these initial tests, further evaluation with a two-hour oral glucose tolerance test (2h-OGTT) is done [2]. A fasting blood glucose is performed after at least eight hours of no caloric intake. A 2h-OGTT is done two hours after a 75 g oral glucose load is ingested.

Per American Diabetes Association guidelines, diagnosis of diabetes mellitus requires an A1C ≥6.5%, fasting glucose ≥126 mg/dL (7.0 mmol/L), 2h-OGTT ≥200 mg/dL (11.1 mmol/L), or a person with classic symptoms of hyperglycemia or a random plasma glucose ≥200 mg/dL (11.1 mmol/L) [3]. Due to the long-term microvascular and macrovascular morbidity associated with diabetes mellitus and the potential to accelerate the course of diabetic nephropathy, these donors should not be allowed to donate.

The next group of donors includes those who do not meet the criteria for diabetes. This group, termed the “prediabetes” group, is defined as having an A1C of 5.7–6.4%, fasting glucose of 100 mg/dL [5.6 mmol/L] to 125 mg/dL [6.9 mmol/L], or a 2h-OGTT value of 140 mg/dL [7.8 mmol/L] to 199 mg/dL [11.0 mmol/L] [3]. In addition to their increased risk of developing diabetes by 5% to 10% per year [4], being in this group is a risk factor for cardiovascular disease. Furthermore, this group has high associations with obesity, hyperlipidemia, metabolic syndrome, or hypertension. According to a 2007 survey of kidney transplant centers across the United States, 49% of programs exclude donors based on elevated fasting blood glucose, however different cutoffs were used to define this [5]. Donors in this group should be counseled appropriately on weight loss, diet modifications, and exercise and should be discouraged from donating.

Donors with a history of obesity, gestational diabetes, or a strong family history of diabetes mellitus should undergo testing with fasting glucose, 2-hr OGTT [2], and A1C. Checking anti-islet cell antibodies in potential donors with a family history of diabetes can be done but its long-term cost effectiveness is unknown.

#### 2.1.2. Hypertension

Hypertension has a well-known association with the development of kidney disease. This needs to be carefully addressed in potential kidney donors as loss of renal mass in a hypertensive patient may increase the risk of parenchymal renal disease developing, or may accelerate the decline of kidney function in those patients in whom other types of kidney disease develop.

The Joint National Committee Seventh Report introduced a classification for hypertension which included the term prehypertension. They defined prehypertension as blood pressure ranging from 120–139 mmHg systolic and/or 80–89 mmHg diastolic. The classification in hypertensives is divided into stage 1 hypertension (systolic blood pressure (SBP) 140–159 mmHg and/or diastolic blood pressure (DBP) 90–99 mmHg) and stage 2 hypertension (SBP greater than or equal to 160 mmHg and/or DBP greater than or equal to 100 mmHg). With this classification system, individuals with prehypertension could be easily identified and counseled due to their increase risk of developing hypertension and cardiovascular disease [6].

For donors, at least two blood pressure readings on two separate occasions should be performed [2]. A donor who has any elevated blood pressure reading is generally sent for 24 hr ambulatory blood pressure monitoring to rule out white coat hypertension or to confirm the abnormal finding.

A donor with a history of hypertension should undergo further evaluation and questioning. If that donor is on two or more medications, he is generally excluded [5]. If that donor is on one medication, then he can continue as a donor as long as he does not have evidence of target end organ damage. According to the 2007 survey, 41% of centers consider donors with well-controlled hypertension on one medication and only 8% will consider donors on two medications [5]. For the donors on one antihypertensive agent, documenting the absence of microabuminuria, left ventricular hypertrophy, or other cardiac disease, dyslipidemia, obesity, or ophthalmologic changes characteristic of hypertension is imperative. In addition, this donor must demonstrate that his blood pressure had been well controlled for at least six months prior to evaluation and that he will have proper followup postdonation for his blood pressure.

Racial variations in the development of hypertension and chronic kidney disease in African Americans and Hispanics have been described postdonation [7]. Because hypertension may have poorer outcomes in African Americans and Hispanics, ethnicity may play a role in excluding a larger proportion of this population of hypertensive potential donors or even these potential donors with an increased risk of developing hypertension.

It is uncertain what to do with donors who are labeled as prehypertensive. These donors are counseled in detail about lifestyle modification and their increased risk of developing hypertension and cardiovascular disease. Of these who opt to donate, postnephrectomy followup by their primary care provider should be suggested.

Individuals with a family history of hypertension are again counseled, as there is a genetic predisposition to developing hypertension. Both paternal and maternal hypertension are strongly associated with the development of hypertension during adult life [8]. Careful counseling and risk analysis should be done before these donors proceed.

#### 2.1.3. Age

Fifty nine percent of centers in the U.S. have no upper age limit. Approximately thirty percent of the centers are using donors over the age of 65 [5]. Advanced age (defined as age above 65) may be associated with decreased recipient graft and patient survival [9]. Biopsies from donors over the median age of 57 years showed age-related nephrosclerosis which may explain these inferior outcomes [10]. Nevertheless, especially due to the shortage of live donors, older donors should not be excluded. Conversations should be undertaken with both recipient and donors in regard to potential long-term disadvantages.

The majority of centers exclude donors younger than 18 years [5, 11]. Young donors should be evaluated on an individual basis, and depending on their level of comprehension of kidney donation, their maturity level, their ability to provide voluntary and valid consent, and their general well being, they should be allowed to donate. Minors and guardians should be informed of the gaps in our knowledge regarding the long-term outcomes in minors with a solitary kidney [12].

#### 2.1.4. Pregnancy

Pregnant patients should not be evaluated. It is not certain when a donor should be allowed to donate post pregnancy [13]. In addition, given recent data on pregnancy and donation, all female donors of childbearing age who are contemplating pregnancy should be counseled on the potential risks of donating. Women postdonation may have an increased risk for gestational diabetes, gestational hypertension, preeclampsia, prematurity, and fetal loss [14]. Another study confirmed the increased risk of preeclampsia and hypertensive disorders of pregnancy [15].

Although there is no clear evidence as to the timing of pregnancy postdonation, it is suggested that women should delay pregnancy until at least two months to a year postdonation to allow and assess for the degree of renal compensation and to assess baseline blood pressures, GFR, and microalbuminuria [16, 17]. A high level of surveillance and monitoring is suggested for all pregnant women postdonation [17].

### 2.2. Renal Issues

#### 2.2.1. Glomerular Filtration Rate

While removal of a kidney in a person with two normal functioning kidneys should not result in progressive kidney failure, a large reduction in nephron mass in a person who already has a decreased baseline glomerular filtration rate (GFR) can have adverse consequences.

The most common approach to estimating GFR is with a 24-hour urine for creatinine clearance. Approximately ninety percent of U.S. transplant centers use this method [5]. Inadequate collection, low protein diet, and other factors may lead to low creatinine clearances in those with actually normal kidney function [13]. If the 24-hour creatinine clearance is borderline, further evaluation with radionuclide methods can be done [2]. These include iodine 124-iothalamate or technetium 99m-diethylenetriamine. The general cutoff for most centers is GFR of 80 mL/min/1.73 m^3^ [5, 11].

#### 2.2.2. Proteinuria

The majority of programs assess proteinuria with a 24-hour urine collection [5, 11]. Other tests that have been used but may not be able to replace the 24-hour protein collection include the spot urine protein to creatinine ratio, spot urine albumin to creatinine ratio, urine microalbumin, or 24-hour urine for albumin excretion [5, 11]. While most programs use >300 mg/day in a 24-hour urine collection as the cutoff, a minority use >150 mg/day as a cutoff [5].

#### 2.2.3. Hematuria

All programs should perform a urinalysis looking for microscopic hematuria. Obtaining any past urinalysis as well as repeating another one would be helpful to determine if the hematuria is transient or persistent. Hematuria is considered significant by one-third transplant centers if there are more than 3 red blood cells/high powered field (RBC/HPF) [5].

If microscopic hematuria is found, an etiology should be determined. Renal ultrasound which is the normal part of the donor workup should be able to reveal most structural causes of hematuria at the level of the kidney. Family history of renal disease including polycystic kidney disease, thin basement membrane disease, or Alport's syndrome or the presence of concomitant proteinuria can suggest an underlying glomerular cause of hematuria in the donor. In such cases, renal biopsy should likely be performed. Dysuria, urinary frequency, and the presence of pyuria can suggest a urinary tract infection. A donor with a family history of renal cell carcinoma or with a strong smoking history should be evaluated for malignancy with a urologic evaluation, urine cytology, and cystoscopy. In men, prostate issues should be ruled out. In women, making sure the urine specimen was not taken at the time of their menstruation is important. African American patients should be screened for sickle cell disease or trait. Asymptomatic nephrolithiasis should be ruled out as well. Almost half of transplant centers will accept donors with >10RBC/HPF if renal and urologic evaluations are negative [5].

A kidney biopsy can identify unexpected pathology and can assist in evaluating the donor. Correlations between a time zero biopsy and donor characteristics have been made leading to the following correlations: interstitial fibrosis and age, tubular atrophy and diastolic blood pressure and proteinuria, arteriolar hyalinosis and serum creatinine and estimated glomerular filtration rate, mesangial increase, and body mass index [18]. Long-term allograft outcomes in donors with these histologic characteristics are still uncertain [19]. More recently, in a study of deceased donors, a donor risk score using ten risk factors was calculated and used to prognosticate the chronic allograft damage index (CADI) using a histologic grading system [20]. This study showed a correlation between donor risk scores, greater degree of histologic lesions, and worse graft outcomes. Whether kidney biopsy should be done in all potential living donors requires further studies, taking into account risk of the biopsy itself, and sampling error. Definite indications may be persistent unexplained microscopic hematuria or proteinuria, or any other uncertainty of a possibility of underlying glomerular disease in the potential donor. If the histopathology is consistent with glomerular disease the donor should be ruled out.

#### 2.2.4. Nephrolithiasis

Nephrolithiasis with development of obstruction of a single kidney can have catastrophic consequences yet a history of nephrolithiasis need not preclude donation as long as certain conditions are met. The number of stones the potential donor has formed in the past needs to be determined. For example, if a donor has a history of nephrolithiasis but the disease has been quiescent for years and there are no abnormalities in metabolic or radiologic testing, then donating can be acceptable [2]. This donor, however, should be informed that his lifetime risk for developing another stone can be 14% at 1 year, 35% at 5 years, and 52% at 10 years [21]. If, however, a donor has had recurrent episodes of nephrolithiasis, he or she should be discouraged from donating. 

The other end of the spectrum includes those who do not give a history of nephrolithiasis but are found to be asymptomatic stone formers. Stones may be noted incidentally while doing screening renal ultrasounds as part of the donor evaluation. In addition, some donors may have a strong family history of nephrolithiasis. These donors can likely be accepted but must be cautioned about their increased risk of developing another kidney stone in their solitary kidney during their lifetime.

### 2.3. Lifestyle Issues

#### 2.3.1. Overweight and Obesity

Potential donors who are either overweight or obese pose a number of issues. Some of these relate to the perioperative period where the risk of complications during this time is increased. However, there are special long-term considerations as well and donation of a kidney in someone who is obese may enhance their risk of eventually developing kidney disease. It should also be noted that the increasing prevalence of obesity may prove to be a substantial limiting factor to rates of kidney donation.

All donors should have their height and weight checked during their initial visit. Body mass index (BMI) is then calculated which is defined as weight in kilograms divided by height in meters squared. Those with BMI between 25 kg/m^2^ and 29.9 kg/m^2^ are considered overweight and those with BMI greater than 30 kg/m^2^ are considered obese. Obesity is further subclassified into class I (BMI of 30.0 to 34.9 kg/m^2^), class II (BMI 35.0–39.9 kg/m^2^), and class III (BMI greater than 40 kg/m^2^). Higher baseline BMI has been shown to be an independent risk factor for end-stage renal disease [22].

The majority of transplant centers exclude potential donors with a BMI over 35 kg/m^2^ [5]. Those with BMI over a 30 kg/m^2^ but less than 35 kg/m^2^ should be counseled on lifestyle modifications especially weight loss. In addition, they should be made aware of the long-term associations of obesity with the development of hypertension, diabetes mellitus, metabolic syndrome, and kidney disease, including end-stage renal disease.

#### 2.3.2. Smoking

Smoking is an area of concern for a number of reasons but is currently not a contraindication to donate. While the postoperative risks of poor wound healing and pulmonary infections in smokers are known [16], the long-term effects of smoking on the kidneys are uncertain. In a recent study, smoking has been shown to be associated with an increased risk of developing proteinuria possibly through glomerular hyperfiltration [23]. On the contrary, in a study of living donors, smoking status was not a risk factor for long-term decline in glomerular filtration rate [24]. With this in mind, due to the negative postoperative risks and extrarenal long-term effects of smoking, donors should be counseled on smoking cessation and advised about the negative consequences of smoking.

#### 2.3.3. High-Risk Behaviors

Donors should have behavioral screening in addition to infectious disease testing. Sexual history and drug history should be examined in detail. They should be interviewed in a confidential manner and should be asked direct questions. Issues of concern are promiscuity; men who have had sex with other men in the previous five years; anyone exposed to or who has had sexual relations with someone at risk for, or with HIV, HCV, or HBV in the preceding year; donors with a history of hemophilia; donors with history of blood transfusions or any human-derived clotting factor concentrates; intravenous, intramuscular, or subcutaneous drug use in the preceding five years; men or women who have had sex in exchange for money or drugs in the previous five years; incarceration [25]. A substantial disadvantage of these donors is the potential to transmit infectious diseases, namely, HBV, HCV, and HIV.

Depending on the situation, donors can be excluded if their risk to the recipient is very high. If they are currently engaging in any high-risk behavior, they should be advised of their obligation to avoid the high-risk behavior. If they cannot avoid it, they should be given the choice to opt out of donating with a medical excuse. Due to the high risk, the recipient advocate and recipient should be made aware of the potential increased risk to the recipient for infectious diseases [26]. Informed consent from the recipient should be obtained and documented in regard to this [26].

It is controversial at this point whether to repeat serology or perform NAT for HBV, HCV, and HIV in high-risk donors two to four weeks prior to transplant. The purpose of NAT would be to detect the disease at the time period between infection and detectability of antibodies. The downside of NAT is that it costly, takes longer to perform and may result in false positives [27].

### 2.4. Psychosocial Issues

A thorough psychosocial evaluation adds to the comprehensive evaluation of the donor. Ideally, this evaluation should be done by a social worker and/or psychiatrist who has experience in evaluating donors. The goal of this evaluation is to evaluate the psychological, social, and emotional stability of the potential donor [12]. In addition, the donor's ability to give informed consent, their underlying motivation to donate, their relationship to the recipient, and any economic or social hardships that the donor may encounter during or after the process are sought after [28].

An in-depth evaluation of the donor's current mental condition should be done. Any donor with a history of any psychiatric disorder, including major depressive disorder, bipolar disorder, generalized anxiety disorder, mood disorder, or any personality disorder should be assessed for any signs or symptoms of instability. A donor should be asked about any childhood or adolescent problems that suggest mental health issues, suicidality, homocidality, inpatient psychiatric hospitalizations, or outpatient psychiatric evaluations. A thorough assessment of any medications that they were taking or currently taking should be done, keeping in mind any medications that could potentially be nephrotoxic. For example, if a donor has been stable on lithium carbonate and cannot be taken off of it due to fear of relapse, that donor should likely not donate [13]. In addition, the evaluator must try to foresee any potential problems that can arise during the postdonation period, and any risk of recurrence. In fragile donors, evaluation and counseling should extend to the donor during the postdonation period to assure psychological well being. Testing focusing on the donor's level of cooperation, eye contact, speech, language, mood, affect, thought process, thought content, memory, orientation, insight, and judgment should all be noted.

Donors should not have any evidence of active drug or alcohol abuse. Generally, a period of at least six months of abstinence is acceptable for a donor [13]. Again, the high-risk donor can be identified at this time as well with a detailed and thorough sexual history and counseled in an appropriate manner. Smoking cessation can be discussed at this evaluation as well.

 The relationship between the donor and the recipient should be established, as well as what is motivating the donor to donate. Any suggestion of coercion or guilt should be further evaluated, making sure that the donor is not threatened in any way and that there is no gain—either financial or personal. In other words, the donor should be making decisions out of his free will. Other donor and family relationships should be addressed, looking for any signs of marital problems, or any hardships that the donor or evaluator can foresee as a result of donation. Evaluation of the donor's social support system must be made [28].

Any potential economic loss should be evaluated. If time off from work during the postdonation period could cause the donor to lose his job or lose major income, the donor should reconsider. Any out-of-pocket expenses for travel, lodging, or miscellaneous expenses related to transplant or inability to obtain sick leave should be discussed with the donor [12, 28]. Education level and socioeconomic status should be evaluated, making sure that these do not play a role in the donation process. These can sometimes be a barrier to informed consent or to the donor's understanding of the entire process [29]. The donor's religious beliefs should be evaluated as well, making sure that the donor is not being motivated by certain beliefs [29].

#### 2.4.1. Obtaining Informed Consent

Donors must demonstrate competence and understanding regarding the donation process and recipient health. He must also receive all the accurate information to make a decision. The donor should demonstrate understanding of the recipient's illness and should understand the other options for the recipient, including deceased donor transplantation or dialysis. He should be able to describe to the best of his knowledge why the recipient needs a transplant. The donor should be able to demonstrate understanding of the nephrectomy including the risks and benefits to the recipient, the risks and benefits to the donor, the postoperative course, the short- and long term-outcomes associated with nephrectomy, the possibility of rejection, and the possibility that the donor may need the sacrificed organ in the future. All preoperative workup, expenses, and postoperative surgical complications and expected outcomes should be fully disclosed to the donor [12].

The donor should demonstrate that he is free of coercion or any outside influence and that his decision to donate is voluntary [12, 13]. The donor must have the capacity to give informed consent. He must have passed the social and psychosocial evaluation with no major issues. The donor must also be made aware of his right to reconsider donation at any point during the evaluation, of his right to opt out with a medical excuse, and of the confidentiality of the donation process [29].

## 3. Donor Assessment to Protect the Health and Safety of the Recipient

### 3.1. Infection

Kidney donation entails the risk of transmission of an infectious disease to a recipient who will be subjected to significant immunosuppression. Hence, the presence of any such infection must be tested for.

All donors must be screened for active and past communicable infections. This testing should include human immunodeficiency virus 1 and 2 (HIV-1 and HIV-2), human T lymphotropic virus type-1 (HTLV-I) and type-2 (HTLV-II), hepatitis A, hepatitis B, hepatitis C, and syphilis. In addition, donors are further tested for cytomegalovirus (CMV), epstein-barr virus (EBV), toxoplasmosis, herpes simplex virus (HSV), and varicella zoster virus (VZV).

Screening for HIV should include serum enzyme-linked-immunosorbent assay (ELISA) for HIV-1 and HIV-2 using U.S. Food and Drug Administration licensed screening tests. Combination HIV-1/HIV-2 tests are available. Screening for Hepatitis B should include qualitative detection of hepatitis B surface antigen (HBsAg) and antibodies to hepatitis B core antigen (Anti-HBc, IgG, and IgM) and to HBsAg (Anti-HBs). Screening for hepatitis C can be with antibody testing to hepatitis C virus (Anti-HCV). Whether nucleic acid testing (NAT) should be done in addition to or in place of the above is unknown and case-dependent. Per Centers for Disease Control and Prevention (CDC) recommendations, all recipients should be counseled that negative tests for HBV, HCV, and HIV do not confer absence of disease, as the donor could have donated after infection but before serocoversion [25]. Syphilis can be screened for with Venereal Disease Research Laboratory (VDRL) or Rapid Plasma Reagin (RPR). HTLV I/II screening is done with ELISA.

Having any of the above active infections would be a contraindication to donate. Having positive testing for HIV, HCV, HBsAg, or anti-HBc, IgM positivity would be a contraindication to donate. Hepatitis B Core Antibody IgG positivity in an HbsAg-negative donor carries a small risk of transmission and such donors should be excluded [30]. Being Anti-Hbs positive is not a contraindication as this could signify vaccination or long-term immunity. Here, history can elicit which is the etiology. Having past infection with toxoplasmosis, CMV, VZV, HSV, or EBV is not a contraindication to donate. Syphilis does not contraindicate a person from donating as the risk of transmission is very low but the donor should be treated prior to donation and the recipient should be treated with penicillin posttransplant [30]. Being HTLV-I-positive should ideally be a contraindication given there could be increased risk for myelopathy and adult T cell leukemia in the immunosuppressed recipient. Given minimal chance of HTLV-I/II disease in the recipient approximately 12 months posttransplant, careful consideration can be given to these donors [31]. Long-term outcomes, however, are not available and one should proceed with caution, after obtaining appropriate informed consent.

Screening for *Strongyloides*, *Trypanosoma Cruzi*, West Nile Virus, Tuberculosis with purified protein derivative testing or the interferon gamma release assay should be done on donors from endemic areas if these diseases are suspected.

### 3.2. Malignancy

Kidney donation entails a potential risk of transmission of malignancy to the immunosuppressed recipient. With careful evaluation of donors, unintentional transmission of cancer should be avoidable. The evaluation of potential malignancy in the donor should begin with age appropriate health maintenance screening. Recommendations per the U.S. Preventative Task Force are as follows: all women who are sexually active and have a cervix or those above 18 years of age should have pap smears. Biennial screening mammography for women beginning at age 50 should be performed. Screening for colorectal cancer using fecal occult blood testing, sigmoidoscopy, or colonoscopy, in adults, should generally be done beginning at age 50 years [32]. In male donors age 50 or above, whether symptomatic or not, prostate screening testing should be performed with digital rectal examination and prostate specific antigen (PSA) [2]. If there is a family history of malignancy, screening should be done at an earlier age after discussion with the donor [2].

 According to the Disease Transmission Advisory Committee (DTAC) Malignancy Subcommittee, donors can be categorized into risk categories including no significant risk, minimal, low, intermediate, high, or unknown risk [33]. Each of these risk categories divides patients according to specific tumor type. Absolute contraindications and high-risk donors include those with a history of lung, breast, renal, or other urologic cancers, melanoma, neuroendocrine tumors, or small cell carcinoma any site of origin, monoclonal gammopathy, choriocarcinoma, testicular cancer, gastrointestinal, or hematologic malignancy such as leukemia or lymphoma [2, 33]. Donors of the high-risk category carry a greater than 10% transmission rate. Minimal risk category patients include those with basal cell carcinoma of the skin, squamous cell carcinoma of the skin, *in situ* cervical cancer, *in situ* vocal cord carcinoma, and certain thyroid cancers [33]. These donors carry a <0.1% transmission risk. Transmission risks are meant to provide a basis from which to suggest levels of concern, as true transmission frequency estimates have not yet been established [33, 34].

 It is advised that every precautionary measure be taken. During organ retrieval, if a suspicious mass or nodule is found, biopsy should be performed and prompt frozen section examination should be done [35].

## 4. Conclusions

The final decision for transplantation should respect the donor's autonomy and decision to donate, the recipient's right to accept, and the respective transplant teams' medical decision making to proceed for living donor transplantation [12]. All parties involved must be free of coercion. The risk and benefit analysis for the donor should be to provide more good than harm. If the opposite is the case, then transplantation should not proceed. The task may be difficult but if done carefully will result in good outcomes.

## Figures and Tables

**Table 1:** Summary of pretransplant donor evaluation.

Donor screening components	Rationale
*Screening to Protect the Donor*	
Medical	
General medical/Cardiac preop assessment	Perioperative and long-term risks
Diabetes Mellitus	Risk of chronic kidney disease, cardiovascular disease
Hypertension	Risk of chronic kidney disease, cardiovascular disease
Age	Risk of chronic kidney disease
Pregnancy	Rick of complications during pregnancy
Renal	
Glomerular filtration rate	Risk of chronic kidney disease
Proteinuria	Risk of chronic kidney disease
Hematuria	Risk of chronic kidney disease
Nephrolithiasis	Risk of kidney disease (acute and chronic)
Lifestyle	
Overweight and obesity	Metabolic, cardiovascular risks
Smoking	Cardiovascular and possible renal risks
Assessment for high risk behaviors	Long-term medical risks
Psychosocial	
Psychosocial and psychiatric issues	Short term and long-term exacerbation
Informed consent	Risks related to not understanding, coercion

*Donor screening to protect the recipient*	
Medical	
Infection	Risk of transmission of infection to recipient
Malignancy	Risk of transmission of malignancy to recipient
Renal	Risk of donor derived kidney disease
Lifestyle	
Assessment for high-risk behaviors	Potential for transmission of an unidentified infectious agent
